# Patient and clinician perspectives on pharmacogenetic testing for antipsychotics

**DOI:** 10.3389/fphar.2025.1689300

**Published:** 2025-10-09

**Authors:** Maria Richards-Brown, Yiran Wei, Rosemary Abidoph, Lauren Varney, Marius Cotic, Stephen Murtough, Daniele Panconesi, Daisy Mills, Alvin Richards-Belle, Noushin Saadullah Khani, Beverley Chipp, Elvira Bramon, Nicola Morant

**Affiliations:** ^1^ Division of Psychiatry, University College London, London, United Kingdom; ^2^ Department of Genetics and Genomic Medicine, Great Ormond Street Institute of Child Health, University College London, London, United Kingdom; ^3^ North London NHS Foundation Trust, London, United Kingdom; ^4^ SideBySide Network, St Pancras Hospital, London, United Kingdom

**Keywords:** pharmacogenetics, mental health, psychosis, antipsychotics, psychiatry, personalized medicine

## Abstract

**Background/objectives:**

Medications to treat psychosis (i.e., antipsychotics) have common and sometimes serious adverse drug reactions and can require several trials before finding a suitable drug and dose. To address this, there is increasing focus on personalizing medicine. Pharmacogenetics investigates how genetic variation influences drug metabolism and response, with recent clinical trials suggesting pharmacogenetic testing can improve remission and reduce adverse drug reactions. Therefore, understanding stakeholder perspectives on acceptability is critical.

**Methods:**

This pilot study is part of ‘GEMS’ (Genetics and Environment in Mental Health Study), which investigates pharmacogenetic testing for psychosis. A participant survey, co-created with patients, was completed by 22 patient-participants, and semi-structured interviews were conducted with 11 clinician-participants who had used pharmacogenetic test reports with patients.

**Results:**

Both patients and clinicians were generally positive about pharmacogenetics, although clinicians saw this as just one component in the multifactorial process of individualized prescribing. Clinicians and patients both suggested a more user-friendly format of the pharmacogenetic report to enhance patient understanding. Some described the reports as promoting more collaborative care, but this was not universal. Clinicians highlighted both retrospective and prospective value in pharmacogenetics providing more certainty through reducing ‘trial-and-error’ prescribing. However, accessibility, understanding, and logistics were identified as potential barriers to implementation.

**Conclusion:**

Among patients and clinicians who have experienced pharmacogenetic testing to inform antipsychotic prescribing, acceptability is good. There is potential for pharmacogenetics to enhance personalized prescribing, but barriers to widespread implementation remain.

## 1 Introduction

Psychosis is a broad symptom category characterized by hallucinations and delusions, which lead to altered thoughts and feelings. It can occur in various mental health disorders, including schizophrenia. Schizophrenia, a severe and chronic psychiatric disorder, affects approximately 1% of the UK population (Bebbington and McManus, 2020).

Current UK National Institute for Health and Care Excellence (NICE) guidelines recommend antipsychotics as the first-line treatment option for psychosis and schizophrenia (NICE, 2014). A range of antipsychotics are available, with differing adverse effect profiles. However, although an estimated 30% of individuals with schizophrenia do not respond to first-line antipsychotics (McCutcheon et al., 2021; Taylor et al., 2025), clinicians do not know in advance which individuals will respond, and which specific antipsychotic at what dose will best suit an individual patient (Davis and Chen, 2004). Therefore, in practice, clinicians (including psychiatrists, nurse prescribers, pharmacists, etc.) often adopt a trial-and-error approach to find a safe and effective antipsychotic prescription.

Treatment failure, adverse drug reactions (ADRs) such as weight gain, lethargy, mental clouding or decreased libido (Angadi and Mathur, 2020), and lack of insight (Kim et al., 2020) contribute to high discontinuation rates and not taking medication as prescribed (Diniz et al., 2023). For example, a systematic review estimated that 56% of patients with schizophrenia were non-adherent to their psychotropic medication (Semahegn et al., 2020). Antipsychotic discontinuation, however, is associated with nearly double the rehospitalization rates compared to maintenance (Vinkers et al., 2024). It is, therefore, unsurprising that patients have mixed views of antipsychotics. Users often see them as ‘the least worst option’, reflecting the challenges of weighing up symptom management and medication side-effects (Morant et al., 2018). Qualitative studies highlight that patients lack information about medication, feel under-involved in medication decisions, and feel their choices about taking medication are limited (Bjornestad et al., 2020; Kaar et al., 2019), often leading to feelings of disempowerment.

Concerns surrounding interindividual variability in antipsychotic response have led to the development of more personalized therapeutic strategies. For example, the Psymatik Treatment Optimizer is a digital antipsychotics optimization tool that weights an individual’s concerns surrounding fourteen side-effects covering thirty-two antipsychotics (Pillinger et al., 2023). Medications are ordered according to an individual’s preferences, thus promoting personalized prescribing. However, this model lacks evidence for its clinical use and remains limited as it does not consider other factors such as age, sex, ethnicity, or genetics.

Pharmacogenetics investigates how genetic profiles influence drug metabolism (pharmacokinetics), as well as other drug-gene interactions (e.g., pharmacodynamics), and thus may influence responses to medications. For example, the cytochrome P450 (CYP) enzyme family plays a prominent role in drug metabolism (Lynch and Price, 2007), and the genes that code for these enzymes are critical in determining individual metabolic capacity. The *CYP2D6* gene, which has over 100 genetic variants (Murphy et al., 2022), encodes the CYP2D6 enzyme, which is responsible for metabolizing approximately 25% of all medications, including most antipsychotics (Ravyn et al., 2013). Other key genes include *CYP1A2* and *CYP3A4*, which code for enzymes involved in the metabolism of antipsychotics, including clozapine and quetiapine (Pardiñas et al., 2019; Menus et al., 2020). Pharmacogenetic variation is highly prevalent, as up to 99.5% of individuals may respond atypically to at least one medication and nearly 24% of people have previously been prescribed a drug for which they are predicted to respond atypically (McInnes et al., 2021). Pharmacogenetics identifies an individual’s genetic variants to infer their metabolizer status and thus contribute to a more person-centered approach to prescribing. For example, ‘poor metabolizers’ have genetic variants that cause reduced or absent enzyme activity, causing slower drug metabolism. The drug then stays longer in the body or at higher plasma levels, increasing risk of side effects. Pharmacogenetic testing may point to a lower dose or alternative drugs to minimize ADRs and maximize efficacy. Conversely, ‘ultrarapid metabolizers’ metabolize drugs faster, meaning that a smaller fraction of active drug reaches systemic circulation, which may affect therapeutic efficacy. As a result, these individuals may be advised to take a higher dose or an alternative medication.

Currently, pharmacogenetic testing is used in oncology to inform prescription of certain cancer medications as toxicity and efficacy can be affected by particular genes (Relling and Dervieux, 2001) and interest in similar approaches in psychiatry is growing. A recent multi-specialty, prospective, randomized clinical trial (PREPARE) across seven countries including the UK found that using a 12-gene pharmacogenetic panel significantly reduced ADRs by 30% (*p* = 0.008), showing the clinical utility of pharmacogenetics in medicine (Swen et al., 2023). In specifically psychiatric patients (i.e., those with schizophrenia, depression, or bipolar disorder), pharmacogenetic testing led to a 34.1% reduction in ADRs (*p* = 0.049), 41.2% fewer hospitalizations (*p* < 0.001), and 40.5% fewer re-admissions (*p* = 0.15) compared to the control arm (Skokou et al., 2024). Similarly, a meta-analysis by Brown and colleagues (2022) reported that patients with major depressive disorder were 40% more likely to achieve symptom remission with pharmacogenetic-guided antidepressant therapy compared to those on antidepressant treatment as usual (*p* = 0.001).

Looking at pharmacogenetics specifically in the context of psychosis, Kang and colleagues (2023) found that, compared to treatment as usual, using an 11-gene panel to guide pharmacogenetic testing significantly reduced Positive and Negative Syndrome Scale (PANSS) scores in Chinese males with schizophrenia (N = 210; 74.2% vs. 64.9%; p < 0.001). However, Jürgens and colleagues (2020) reported no difference in antipsychotic drug persistence (an integrated measure of treatment failure and adverse drug reactions) between Danish patients with schizophrenia who were offered CYP-testing (*CYP2D6* or *CYP2C19*) and those given structured clinical monitoring. The success of Kang and colleagues’ study may be due to using a gene panel, but, nevertheless, the evidence-base for whether pharmacogenetics can optimize prescription of antipsychotics is currently mixed. A recent systematic review of pharmacogenetic testing in psychosis suggests the approach is promising but highlights limitations such as lack of diversity in participants and modest sample sizes (∼400 per study), which could also explain inconsistencies in the literature (Saadullah Khani et al., 2024).

Nonetheless, evidence-based clinical guidelines, such as those from the Clinical Pharmacogenetics Implementation Consortium (CPIC) and the Dutch Pharmacogenetics Working Group (DPWG), now advise genetically-informed prescribing in areas such as cancer, cardiovascular health, and mental health (Muldoon et al., 2024; Hulshof, 2023). The USA Food and Drug Administration (FDA) is also increasingly incorporating pharmacogenetics into drug labels (FDA, 2022).

With increasing use of and evidence for the clinical utility of pharmacogenetics, it is crucial to consider acceptability to stakeholders (both patients and clinicians). A recent review found that the majority of psychiatric patients believed in the utility of pharmacogenetics and, if offered, would undergo pharmacogenetic testing themselves (Tamaiev et al., 2023). Another systematic review of clinicians’ and patients’ views on the implementation of pharmacogenetics in psychiatry reported lack of knowledge, costs of genetic testing and clinicians’ time to deliver the tests as perceived barriers, but optimism about pharmacogenetics leading to precision medicine, reducing ADRs, and becoming a routine practice (Jameson et al., 2021). However, this review did not include any UK studies. A recent survey of the UK public explored opinions on pharmacogenomics in general, not limited to psychiatry, and found that 85% of adults believe the National Health Service (NHS) should offer pharmacogenetic testing where relevant (Magavern et al., 2025). This appears increasingly feasible given that the costs of pharmacogenetic testing continue to decline (Berndt et al., 2019), and there is a body of literature indicating the cost-effectiveness of pharmacogenetic testing for antipsychotics (Herbild et al., 2013; Kurylev et al., 2018; Carrascal-Laso et al., 2021; Saadullah Khani et al., 2024).

Within these reviews, however, few studies recruited patients or clinicians who have themselves experienced pharmacogenetic testing. Virelli and colleagues (2023) found a positive outlook among patients whose psychotropic prescriptions had been guided by pharmacogenetics, with improved confidence in making medication changes. However, Liko and colleagues (2020) reported mixed opinions in patients with depression, with some experiencing pharmacogenetic testing as helpful and others feeling that they did not receive clear prescribing recommendations from their report. Moreover, these studies were conducted in Canada and the US, and given the differences compared to the UK healthcare system, it is essential to gather opinions from UK patients and clinicians to inform UK policy and guidelines.

Pharmacogenetic information may also impact the dynamics in prescribing decisions. Provided that pharmacogenetic results are shared and explained, patients may become better informed about their treatment, potentially enabling them to be more involved in medication-related decisions. Discussion of pharmacogenetic test results between clinicians and patients may encourage a more collaborative approach to drug selection and dosing, thus moving towards a shared decision-making model in which all participants are informed, involved, and influential in treatment decisions (Stacey et al., 2015). Alternatively, if pharmacogenetic test results are used by clinicians without explanation, there may be little or no change in these dynamics. Therefore, it is important to consider not only whether pharmacogenetic tests are used, but how they are used in order to understand their impact and acceptability to patients and prescribers.

In this pilot study we explore UK mental health patient and clinician perspectives on pharmacogenetic testing, focusing specifically on those who have recently received or delivered pharmacogenetic test results. We aim to understand both patient and clinician experiences of pharmacogenetic testing, its impact on psychosis treatment, and opinions on its implementation in routine care.

## 2 Materials and methods

### 2.1 Setting

This research is a part of the broader ‘Pharmacogenetics: Genetics and Environment in Mental Health’ study (GEMS, IRAS ID: 193707). This multi-site, prospective study is conducting reactive (occasionally pre-emptive) genotyping of *CYP1A2*, *CYP2D6*, *CYP2C19* and *CYP3A4* to guide prescribing of psychotropic drugs in patients with psychosis, following evidence-based CPIC or DPWG guidelines (Bousman et al., 2023; Beunk et al., 2024). GEMS is investigating how this pharmacogenetic intervention influences quality of life and adverse drug reactions (Varney et al., 2024). The current study explored views on using pharmacogenetic testing to guide prescribing decisions using semi-structured interviews with clinician-participants and a survey with patient-participants. Previous work with general medicine clinicians (Just et al., 2017) and forthcoming findings from GEMS (Panconesi et al., 2024, *in press*) have already reported survey data on clinician attitudes to pharmacogenetic testing, both in general and specifically for antipsychotics. To build on those findings, we have opted for more in-depth interviews with clinicians. In contrast, patient views within GEMS have not yet been explored, so a survey was considered the most efficient method to initially capture a broad range of perspectives. Ethical approval has been provided by the NHS Health Research Authority (REC reference 19/LO/1403).

### 2.2 Clinician interviews

#### 2.2.1 Sample

Clinician- and patient-participants in GEMS are henceforth referred to as ‘clinicians’ and ‘patients’. We consulted our collaborators from the SideBySide Network, a team of people with lived experience of mental health problems including psychosis, about terminology. Since the majority preferred the term ‘patient’, this term was chosen, although with acknowledgement that this is debated in the field of mental health (Priebe, 2021).

Clinicians in GEMS were consultant psychiatrists, general practitioners, pharmacists, junior doctors, nurse prescribers, advanced clinical practitioners or other clinicians who prescribe antipsychotic medicines. They were working in the UK’s NHS and willing to order pharmacogenetics tests, discuss the results with patients and consider the pharmacogenetics results in their prescribing. It should be noted that PGx testing is not yet available nationally within the UK; at present, initiatives such as the GEMS study represent some of the first opportunities for both patients and clinicians in UK mental health services to experience PGx-guided prescribing. To partake in the optional semi-structured interview, clinicians must have received at least one pharmacogenetics report that showed actionable information (i.e., minor or major prescribing considerations indicated by the report) and discussed it with their patients.

In order to access a variety of clinician voices, sampling was purposive, aiming to include those working in various NHS services across England. A total of 30 clinicians were approached via e-mail (YW; RA) to ask whether they wanted to participate in the clinician interviews. Thirteen initially agreed, although two did not respond further when arranging the interview. Three declined participation (one noting lack of time, one giving no reason, and one noting that they had not yet discussed a pharmacogenetic report), and the remaining clinicians did not reply. Clinicians consented to the interview being audio-recorded and transcribed, and for anonymized quotations to be used for a publication.

#### 2.2.2 Design

The clinician interview topic guide (see Supplementary Material) was designed to explore clinicians’ experiences and views about (a) the interpersonal process of discussing pharmacogenetic reports with patients, (b) impact on prescribing decisions and treatment outcomes, and (c) perceived value, feasibility, acceptability, and implementation barriers. The interview was piloted and refined slightly during early stages of interviewing to elicit more detailed responses.

#### 2.2.3 Data collection

Semi-structured interviews lasted between 30–45 min and were conducted using video calls on Microsoft Teams. The interviewer (YW) was not involved in any other aspect of the study and had no prior interaction with the clinicians. The GEMS study manager, who had prior contacts with clinicians, attended the meetings in a supervisory role with their camera turned off. Following each interview, the interviewer wrote reflective notes summarizing main findings and reflecting on the interview process. These were used as contextual reference points in data analysis.

Data on sex was collected as part of the GEMS study, but other demographic variables (e.g., age or ethnicity) were not collected for participating clinicians.

#### 2.2.4 Data analysis

Clinician interviews were recorded, transcribed, and analyzed using thematic analysis within NVivo software by YW. Analysis was guided by a six-stage process (Braun and Clarke, 2006). Following familiarization, initial codes closely captured interview content and were refined through reflection on their meaning. Connections between codes were progressively made leading to the development of themes and sub-themes. This process was iterative as coding progressed and understandings developed, involving processes such as merging or dropping sub-themes, and re-naming themes. This process was facilitated by documentation using memos, and discussions with other team members throughout the analytic process.

### 2.3 Participant survey

#### 2.3.1 Sample

Patients were 18 years or older, had an ICD-10 diagnosis of a “psychotic disorder” (ICD-10 codes F20 to F31), and were taking or planning to take an antipsychotic medicine. To complete the optional survey, patients had to have had their pharmacogenetic intervention (i.e., their genetic report discussed with them). Patients consented to their survey responses being transcribed and for anonymized quotations to be used for a publication.

Patients across all 12 NHS sites of the GEMS study were offered the option of completing the participant survey. Authors personally approached the patients within the Camden and Islington Boroughs of the North London NHS Foundation Trust site. Of those approached, 60% agreed to complete the participant survey. No follow-up questions were asked to ascertain why patients did not agree to participate.

#### 2.3.2 Design

The research team collaborated with the SidebySide Network to design the survey. Three meetings between November 2023 and February 2024 brought together PPIE contributors and researchers. A draft was shared with colleagues for feedback. The survey was developed specifically for this study to capture patient perspectives rather than to measure predefined constructs using a validated instrument. It was therefore not a validated tool, but rather a bespoke, exploratory measure designed to elicit a broad range of views. The survey aimed to gather patients’ (a) reasons for joining GEMS, (b) experience of involvement in medication-related decisions, (c) understanding of pharmacogenetics, and (d) perceived barriers to using pharmacogenetic testing. The final participant survey took 10–15 min to complete and comprised 18 questions requiring responses in Likert scales, multiple-choice, yes/no, and open-ended formats (see Supplementary Material).

#### 2.3.3 Data collection

Patients completed the survey with M.R.B. and R.A. either at the end of an online follow-up session in the GEMS study 3 months after discussing their genetic report, or in an additional Microsoft Teams call. All participants provided informed consent prior to participation. Responses were recorded verbatim, and automated transcripts were obtained for most. Data was stored in Qualtrics. Data collection for the present analysis occurred between February 2024 and February 2025. Data on sex, age, educational attainment, ethnicity, diagnoses, and medications were collected as part of the GEMS study. Ethnicity was self-reported using standard UK Census categories (White; Black, Black British, Caribbean or African; Asian or Asian British; and Mixed or multiple ethnic groups).

#### 2.3.4 Data analysis

Qualitative and quantitative analysis was conducted as the survey included both open- and closed-response questions. Descriptive statistical analysis was performed on the Likert and multiple-choice items using RStudio (Version 4.4.0 2022.12.0). Quantitative variables are reported as averages (both mean and median values), standard deviations, and ranges. Descriptive statistics evaluated further pharmacogenetics’ role in shared decision-making and the comprehensibility of GEMS. Responses to open questions were content analyzed and illustrative quotes were extracted.

## 3 Results

Data was collected from 11 clinicians and 22 patients. Clinician characteristics are presented in Table 1 and patient demographics presented in Table 2. Results are reported to two decimal places.

**TABLE 1:** Clinician characteristics.

Clinician ID	Job title	Location of NHS trust	Type of service	Number of patients with pharmacogenetic reports	Sex
1	Consultant Psychiatrist	London	Rehab and Rehabilitation	11	F
2	General Practitioner	South-west England	General Practice	1	M
3	Consultant Psychiatrist	London	Adult Community Mental Health Service	5	F
4	Consultant Psychiatrist	London	Research Team	15	F
5	Consultant Psychiatrist	London	Rehab and Rehabilitation	3	M
6	Consultant Psychiatrist	London	Community Team	6	F
7	Consultant Psychiatrist	London	Community Team	3	M
8	Consultant Psychiatrist	London	Inpatient Ward	9	M
9	Consultant Psychiatrist	South-west England	Unknown	6	M
10	Consultant Forensic Psychiatrist	Midlands	Inpatient Forensic Ward	5	M
11	Consultant Psychiatrist	South-west England	Adult Community Mental Health Service	6	M

**TABLE 2:** Patient demographics.

Sample characteristics	n	%	M	SD (±)	Range
Sex					
Male	10	45.00			
Female	12	55.00			
Age (years)	22		38.59	12.36	22–65
Highest level of education					
Secondary education	1	4.55			
Tertiary (e.g., apprenticeships)	11	50.00			
Further education (university)	6	27.30			
Postgraduate	4	18.20			
Years in Education	22		15.25	2.07	12–20
Ethnicity					
Black, Black British, Caribbean or African	1	4.55			
Asian or Asian British	3	13.60			
Mixed or multiple ethnic groups	1	4.55			
White	17	77.30			
Primary diagnosis					
Schizophrenia	3	13.60			
Bipolar disorder	6	27.30			
Personality disorder	1	4.55			
Other psychotic disorder	10	45.50			
Other mood disorder	2	9.09			
Antipsychotic taken					
Aripiprazole	3	13.64			
Clozapine	1	4.55			
Olanzapine	3	13.64			
Quetiapine	1	4.55			
Other Antipsychotic	5	22.72			
Polypharmacy	4	18.18			
None	5	22.72			
Duration of Illness (years)	22		12.05	9.99	0–28
Site recruited					
South East England	7	31.82			
Inner London	15	68.18			

Abbreviations: *n*, number of participants; %, percentage of sample; M, mean average; SD (±), standard deviation.

*Definitions:* Under Antipsychotic Taken, “Other” includes the following antipsychotics: risperidone, zuclopenthixol, amisulpride, paliperidone, lurasidone, flupentixol, haloperidol, cariprazine, and promazine. “Polypharmacy”: the patient is taking 2 or more of these antipsychotics. “None”: the patient is being considered for an antipsychotic.

Results are presented in the following 6 sections in which the responses of clinicians and patients are combined to highlight areas of similarity and difference in patient and clinician perspectives:

−3.1. Patient Reasons for Participation
−3.2. General Views on Pharmacogenetics
−3.3. The Pharmacogenetic Report
−3.4. Patient-Clinician Interactions
−3.5. Perceived Clinical Utility of Pharmacogenetics
−3.6. Barriers to Implementation

### 3.1 Patient reasons for participation

In response to the initial survey question about why they took part in the GEMS study, patients more commonly reported personal interest relating to unsatisfactory prior experiences of antipsychotics. One patient shared, “I was curious … I have been on various types of drugs, and none of them have really helped” (Patient-16), while another said, “I have not had much success with medication, so the study intrigued me” (Patient-14).

Five participated on the recommendation of healthcare professionals, whilst three expressed a desire to contribute to research. For example, one participant saw the study as an opportunity to “give a little bit back” (Patient-10).

### 3.2 General views on pharmacogenetics

Patients overall spoke optimistically about pharmacogenetics, with one describing GEMS as likely to “help so many people in the future” (Patient-3). Clinicians had similar impressions that patients felt positively about pharmacogenetics:

“They're all glad to have done it and think it's got value in use. Yeah, no one, I do not think has been negative about it.” (Clinician-11)

Clinicians emphasized the complexities of prescribing and drug interaction, and several made the point that not every aspect of how a medication impacts on an individual is reflected in the pharmacogenetic report. They felt that prescribing decisions need to attend to patients’ holistic needs beyond how they metabolize medication:

“You need to think about someone’s social situation […] and the rate of metabolism is one factor which I think often is probably quite small compared with other matters.” (Clinician-2)

Several clinicians emphasized that being aware of pharmacogenetic variations does not guarantee improved treatment outcomes, but may contribute to understanding how one aspect of medication works:

“I think it's a good thing to do, but it's not necessarily gonna kind of come up with this Holy Grail solution.” (Clinician-11)

Echoing these comments, one patient stated that “a lot of people are just trying to function, so it's [pharmacogenetics] not a priority (Patient-20).

### 3.3 The pharmacogenetic report

#### 3.3.1 Feelings about the genetic report

Fifteen out of twenty-two patients requested a copy of their genetic report with the predominant motivation being to aid personal understanding. Patients expressed that they wanted “to try and understand the relationship between enzymes and medication” (Patient-15) and “to know what was happening in my body” (Patient-8), highlighting the personal nature of this information.

For clinicians, the report helped them choose a well-tolerated medication with more certainty and confidence due to “science determining and telling me that I should” (Clinician-4).

“[The pharmacogenetic report is] useful in more logically predicting treatment outcomes, potential side effects, and probably most importantly, trying to get dose calculations early on, escalating the doses or starting with more cautious doses.” (Clinician-7)

#### 3.3.2 Understanding of the genetic report

Patients reported a moderate understanding of the genetic report on a scale of 1–10, where 1 indicates no understanding and 10 indicates complete understanding (*n* = 14; M = 7.29, SD = ±3.15; Median = 8). Clinicians found that understanding varied amongst patients, depending on their intellectual functioning:

“I think it's tailoring the amount of information to patients that they want and asking them” (Clinician-10)

However, both patients and clinicians suggested that the complex format, technical language and presentation of the report “could be more user-friendly” (Patient-20):

“…only one person was maybe able to kind of fully really read it so I think maybe you may need to be more user friendly”(Clinician-5)

Participants suggested that “a glossary” (Patient-20) or “summary highlighting key points” (Clinician-7) could be beneficial in helping both patients and clinicians understand the report more independently.

### 3.4 Patient-clinician interactions

Participants among both patient and clinician samples described how the pharmacogenetic report changed the dynamics of clinical consultations, but this was not universal. One patient stated how “this [genetic testing] can make prescribing more bespoke and thoughtful” (Patient-20), demonstrating how pharmacogenetic test reports can initiate person-centered conversations with healthcare professionals and potentially increase patients’ feelings of involvement in medication-related decisions.

“I think definitely going forward it can [make consultations more collaborative] because you're involving that individual and the individual’s not frustrated that you're trying, you know, 3 medicines before 1 works” (Clinician-4)

“The patients report feeling more empowered, better informed.” (Clinician-10)

When asked whether they think pharmacogenetic testing can help with shared decision-making about medications (Q5), 15 patients indicated a strong belief in its utility, 4 expressed a moderate belief, and only 3 were uncertain about its impact. Clinicians tended to agree that the personalized approach to treatment helps make prescribing ‘feel more collaborative’ (Clinician-7).

“I quite enjoyed that having this pharmacogenomics information helps you personalize this information, you know beyond the guidelines.” (Clinician-3)

However, one clinician, while agreeing that the report could improve the perceived legitimacy of prescribing decisions, had not noticed any change in patients’ attitudes in practice (Clinician-8).

### 3.5 Perceived clinical utility of pharmacogenetics

#### 3.5.1 Addressing empirical prescribing

The current empirical (trial-and-error) approach to prescribing antipsychotics was noted by both patients and clinicians. One patient shared that they were “surprised by how unscientific psychiatry is” (Patient-20), and a clinician admitted that “recommendations can be quite blind” (Clinician-10) and that patients were not always given a rationale behind prescribing decisions. Some clinicians believed pharmacogenetics could provide a stronger scientific basis for prescribing:

“So, previously we've been treating blindly, whereas now we've got a little bit more intelligence to support prescribing decisions.” (Clinician-4)

#### 3.5.2 Change in medications

Six patients reported medication changes following their pharmacogenetics test. These could be changes to antipsychotics, but also to other medications commented on in the pharmacogenetic report, such as antidepressants or pain medications. For example, one patient wished to increase their dosage of Olanzapine, as the “25 mg was not working” (Patient-9). The pharmacogenetic report provided the evidence their doctor needed to increase the dose beyond the UK licensed limit of 20 mg/day to 30 mg/day as per licensing in other countries (Medicines Complete, 2023). This highlights how patients may view pharmacogenetics as a tool to inform dosage adjustments. Alternatively, another patient reported switching “from Diazepam to Lorazepam” (Patient-8) following the pharmacogenetic report.

Clinicians also reported making changes partly informed by the genetic report:

“We changed her treatment to valproate and quetiapine. And I think I did that partly guided by the report, because quetiapine came out to be the appropriate drug as compared to haloperidol and risperidone.” (Clinician-3)

Of the patients whose medication changed, three were reasonably happy, two very happy, and one neutral, with none expressing a negative view. However, one clinician observed that the pharmacogenetics results did not always match clinical presentation:

“So there were medications that had been relatively well tolerated, but actually they were a poor metabolizer and you maybe would have expected them to have had worse side effects.” (Clinician-11)

Similarly, one patient stated “I am not unhappy with the study, just the changes made” (Patient-14). Though fluoxetine was predicted to suit their genetic profile, they reported, “I have not seen any positive changes”, demonstrating a discrepancy between their expectation and the treatment outcome.

#### 3.5.3 Retrospective utility

For fifteen patients, the pharmacogenetic report did not indicate a change in medication, with one patient stating the report “showed current medication is the optimum one for me” (Patient-16). No change is needed when patients metabolize their current medications well, but the report was deemed useful to confirm medications’ continual use:

“It’s always relevant. I think it’s relevant regardless of the fact that my patients were already on medication that were able to tolerate well.” (Clinician-6)

Furthermore, clinicians described how pharmacogenetic information can validate previous clinical decisions, which one clinician described as confirming their “clinical hunch” (Clinician-3).

“I know for one person I definitely reduced the dosing because they were a slow metabolizer and were not tolerating the medicine very well” (Clinician-4)

Equally, patients found that pharmacogenetics could explain intolerability to previous medications. One patient recalled thinking that their medication failure “is just in your head” but the pharmacogenetic report demonstrates “in black and white … why they were not working” (Patient-16). These case examples illustrate the retrospectivity utility of pharmacogenetics.

#### 3.5.4 Predictive utility

The pharmacogenetics report was also considered useful for potential future medication changes. One clinician explained that knowing a patient was a rapid metabolizer helped them create “an advanced plan” in case of relapse, involving a quicker prescription of a higher dose of venlafaxine (Clinician-7). Alternatively, the report can inform future clinicians, if a patient is re-admitted:

“Whenever there’s a need for prescribing any psychotropic medications, ask your GP or the prescriber to consult this report and prescribe something of which you are a normal metabolizer.” (Clinician-9)

This also boosted patients’ confidence regarding the future: “I feel very safe knowing the information I do … in terms of any future prescribing of medication” (Patient-3), suggesting that pharmacogenetic reports can have long-standing clinical value. As many patients had been on antipsychotics for years and had reached suitable medications through the usual empirical prescribing, clinicians felt the pharmacogenetic report would be most valuable for first-time patients:

“I think it would be most valuable earlier on in people’s journey […] lots of people are doing it after kind of a few years of trying different treatments. Umm, so it would be more valuable to do earlier on.” (Clinician-11).

One patient wished they “could have had a study like that done on me years ago. I could have saved myself years” (Patient-16), after having spent 16 years on several less suitable medications.

### 3.6 Barriers to implementation

#### 3.6.1 Accessibility

The most reported barrier by patients was accessibility. Physical health challenges were noted, such as a patient struggling “to provide a sample … if they are house bound” (Patient-1). To address this, another patient suggested “a team could go to these participants” (Patient-2). In addition, the complexity of genetic information was noted, with one patient commenting that “the language is quite advanced”, making it difficult for those with complex needs to understand (Patient-8). Another noted that some individuals “might not understand clearly what the benefits are” (Patient-3), potentially deterring them from testing.

Clinicians similarly raised lack of awareness as a barrier since many people do not know that pharmacogenetic testing can be done. One clinician suggested:

“If the public or the population suddenly all raise and say, ‘oh, we want this test before prescribing medication’, probably that will put the government under pressure a little bit.” (Clinician-9)

Concerns also emerged about healthcare professionals’ understanding of pharmacogenetics. One patient stated, “my review was with the GP, and they did not really understand it” (Patient-8). Clinicians acknowledged a lack of knowledge and training for themselves and other members of their team in interpreting pharmacogenetic reports:

“We do not get taught pharmacogenomics at all as a part of our postgraduate training” (Clinician-3)

The availability of support for clinicians was raised by some, with suggestions that it would be desirable to have someone “offering support that they can reach out if they wanted somebody to guide them through that report” (Clinician-4). In GEMS, clinicians received help from the research team to interpret the genetic results.

#### 3.6.2 Patient consent

Many clinicians expressed concern about obtaining consent due to patients’ concerns or illness-related paranoia around genetic testing:

“You'll get some patients who will not want it. Fear of being kind of genetically profiled … or something like that.” (Clinician-11)

This was reiterated by a patient who suggested people may feel “nervous about providing DNA/blood” (Patient-19). One clinician suggested that saliva swabs are generally more acceptable than blood samples, as they are “less painful” and “less invasive” (Clinician-8). Both options were offered in GEMS.

#### 3.6.3 Logistical barriers

Clinicians were aware that the cost of pharmacogenetics needed to be considered:

“Cost is an element I think needs to be thought about. We have to be pragmatic.” (Clinician-7)

Some clinicians noted that more empirical evidence for the clinical utility of pharmacogenetics could help secure NHS funding and improve access. Practical challenges were also raised: clinicians raised issues of having sufficient “manpower for taking the bloods and getting it sent off to the lab” (Clinician-8) and a need to “develop more laboratories to do the test” (Clinician-9). Report turnover time was another barrier. Early GEMS participants experienced long delays in receiving reports, even “months and months really right at the beginning” (Clinician-1), forcing clinicians to initiate treatment without pharmacogenetic guidance:

“I do not have the luxury to wait for 3 months for a report to make my clinical decisions.” (Clinician-3)

Time constraints in clinics further inhibited implementation for some:

“We’re sometimes hurried because we’ve got four or five patients waiting in clinic […] it’s a little bit of a shame that we do not have that time to explain everything.” (Clinician-4)

This may consequently hinder patients’ understanding, as clinicians felt the reports were not always adequately explained. Relatedly, some patients felt that discussions with their clinicians were inadequate: “he [GP] did not really understand [the genetic report], did not have time to properly go through it” (Patient-20).

## 4 Discussion

This study investigated UK patients’ and clinicians’ attitudes towards pharmacogenetics, specifically those who recently underwent pharmacogenetic testing. Whilst there were nuances in opinions, the overall perception was largely positive, corroborating previous observations that patients are optimistic about pharmacogenetic testing (Virelli et al., 2023; Liko et al., 2020). Greater levels of patient confidence were identified in this study than previously found (Liko et al., 2020), which may reflect subsequent advancements in pharmacogenetics (Singh, 2023).

### 4.1 Pharmacogenetics and collaborative care

The majority of patients reported a strong belief that pharmacogenetic testing can enhance joint decision-making. Several patients suggested that pharmacogenetic reports prompted more patient-centered conversations, helping them feel more informed about their medication-related decisions.

Several clinicians similarly observed patients feeling more empowered following pharmacogenetic testing. However, this perspective was not universal, with some clinicians expressing more neutral views, reflecting variability in opinions of pharmacogenetic testing. Nevertheless, overall attitudes remained optimistic about its potential in enhancing collaborative care.

While these preliminary findings suggest a positive impact of pharmacogenetic testing on patient-clinician interactions, further research with larger and more diverse samples is needed to fully explore how pharmacogenetic testing influences patient-clinician relationships.

### 4.2 Perceived clinical utility of pharmacogenetic testing

Survey and interview data highlighted the clinical utility of pharmacogenetic testing across all stages of care. Several patients and clinicians reported that the pharmacogenetic report helped identify more suitable drugs or dosages. Clinicians suggested that pharmacogenetics might be particularly beneficial for newly presenting patients by reducing unnecessary medication changes. This aligns with findings that 80% of patients in Early Intervention in Psychosis (EIP) services underwent antipsychotic changes that are known substrates of CYP2D6, yet pharmacogenetic testing is not routinely available in these settings (Yeisin et al., 2017). Earlier implementation of pharmacogenetics could improve initial treatment experiences by reducing trial-and-error prescribing, thus minimizing adverse drug reactions and poor therapeutic responses (Patel et al., 2017). Future research should compare the experiences of antipsychotic-naïve patients with long-term antipsychotic users to assess the impact of early pharmacogenetic testing.

The retrospective utility of pharmacogenetics was also evident. Both this survey and Liko’s research (Liko et al., 2020) found that pharmacogenetic results helped explain previous treatment responses, even without leading to present medication changes. Patients described feeling better able to understand their treatment history and validated in their adverse experiences with unsuitable medications.

Patients and clinicians also commented on the prospective utility of pharmacogenetics in guiding future prescribing, particularly when medication changes occur later in treatment. Generally, the pharmacogenetic report was viewed as valuable throughout the care process, not only at the point of testing.

However, clinicians consistently noted that pharmacogenetics is just one piece of a complex clinical puzzle. So, while broadly optimistic about its retrospective, current, and prospective clinical utility, they emphasized the need to integrate pharmacogenetics with other biological, psychological, and social factors. Furthermore, managing patient expectations is crucial to avoid potential disappointment, as there were some reported instances where the pharmacogenetic report did not align with clinical presentations.

Overall, this sample viewed pharmacogenetics positively, as a tool to optimize prescribing, guide dosing, and reduce adverse drug reactions (Swen et al., 2023; Skokou et al., 2024).

### 4.3 Barriers to pharmacogenetic testing

Accessibility was the most commonly identified barrier, with both patients and clinicians suggesting that some individuals may be unable to provide a sample (i.e., due to a physical inability) or struggle to understand the complex genetic information. However, many patients reported high levels of comprehension of the report and their discussions with clinicians, with some expressing a desire to learn more about their genetics. Equally, no clinician in this study reported marked difficulty in explaining genetic reports to patients. Therefore, while comprehension may vary, researchers and healthcare professionals should be careful not to underestimate what patients can and want to comprehend regarding their genetics and medication.

A few clinicians alluded to the inequality in access that may be due to the public not even being aware of pharmacogenetics’ existence, thus not demanding its implementation in the NHS. Therefore, for pharmacogenetic testing to be more widely adopted, efforts must be made to make information more available to potential users. Additionally, as both patients and clinicians suggested, comprehensibility of the pharmacogenetic report should be ensured by developing a more user-friendly version of the genetic report.

Similar to previous studies (Virelli et al., 2023), the present study also identified concerns about the ability of healthcare professionals to interpret pharmacogenetic reports. Inadequate clinician training in pharmacogenetics is not isolated to patient perceptions. A recent survey, yet to be published, echoes these semi-structured interviews that many clinicians admit a lack of confidence and knowledge in pharmacogenetics (Panconesi et al., 2024, in press). As pharmacogenetics is a new field in mental health, this lack of training is a global issue, not just in the UK (Just et al., 2017). Moreover, as pharmacogenetic information becomes more accessible to the public, through direct-to-consumer genetic testing (DTC-GT) which bypasses the need for healthcare professionals (Tafazoli et al., 2021), clinicians will increasingly need to respond to DTC test results in their practice. In the long term, continuous professional development (CPD) for clinicians, and psychoeducation for patients, may be needed.

Similar to previous studies (Tamaiev et al., 2023; Jameson et al., 2021), several clinicians identified costs and lack of research evidence as barriers to implementing pharmacogenetics. However, pharmacogenetic tests are becoming cheaper and more widely accessible (Berndt et al., 2019), and there is a growing body of evidence supporting their cost-effectiveness (Saadullah Khani et al., 2024; Virelli et al., 2021; Herbild et al., 2013). For example, Morris and colleagues reported that, out of 108 studies investigating 39 different drugs, 71% demonstrated pharmacogenetic testing was either cost-effective or cost-saving (Morris et al., 2022). This expanding evidence base on the health economics of pharmacogenetic testing should help alleviate concerns among clinicians. Moreover, pre-emptive pharmacogenetic testing (before any treatment starts) holds the potential to be even more cost-effective, especially because a single test can be used to inform prescribing over the years.

### 4.4 Strengths and limitations

To our knowledge, this is the first UK study to explore the attitudes of both patients and clinicians with direct experience of pharmacogenetic testing for antipsychotics. A crucial strength of the participant survey is that it was co-designed with people with lived experience of psychosis–an approach lacking in previous studies (Virelli et al., 2023; Liko et al., 2020). This ensured the survey was comprehensive, sensitive, and addressed relevant concerns and experiences of those affected by psychosis. Moreover, the survey’s brief format (approx. 10 min) helped minimize fatigue and encourage completion.

Nonetheless, limitations remain. Both the survey and interviews were retrospective. To mitigate recall bias, the survey was administered at the end of a 3-month follow-up period, or shortly thereafter. Similarly, clinicians were encouraged to revisit reports during interviews. The survey and interview samples were small (N = 22; N = 11), especially given GEMS has now recruited over 600 patient-participants and over 200 clinician-participants. Future research should aim to collect data about acceptability and concerns from all participants in pharmacogenetics studies to get a clearer picture. Additionally, the number of patients from minority ethnic backgrounds was insufficient to fully capture diverse perspectives. Although the broader GEMS sample is diverse, with 36% of the participants coming from minority ethnic backgrounds, our subsample was too small to reflect that.

Sampling bias is also possible, as clinicians who were willing to be interviewed may be more comfortable with, and supportive of, pharmacogenetics in the first place. Similarly, the patient sample in GEMS likely consists of individuals already engaged with their medication, thus may not represent the wider population of individuals taking antipsychotics. Severely unwell patients, for example, are less likely to participate in research (Jameson et al., 2024). Additionally, those who agreed to give an extra 10 minutes of their time for the survey may have had more positive experiences with the GEMS study, potentially skewing the results. Therefore, while the study provides valuable insights, caution should be exercised when interpreting the findings, and further research with larger, more diverse samples is required to validate and expand upon these results.

### 4.5 Clinical implications

First, the positive reception of pharmacogenetics among patients and clinicians, combined with the current evidence supporting pharmacogenetic testing for psychosis (Kang et al., 2023), suggests the importance of allocating resources to this area.

Secondly, the lack of confidence in healthcare professionals’ understanding of pharmacogenetics highlights a need for further training. Guidance in interpreting genetic reports, communicating genetic information, and applying it to prescriptions is essential. While foundational pharmacogenetics knowledge should be incorporated in medical curricula, traditional educational approaches may not keep up with the field’s rapid developments. Instead, up-to-date online resources, such as GeNotes (e.g., Genomics Education, 2024) or the Pharmacogenomics Knowledgebase (Whirl-Carrillo et al., 2021), could be more widely promoted to increase awareness and prepare healthcare professionals to deliver high-quality pharmacogenetic testing.

Finally, findings illustrate the importance of managing patient and clinician expectations. Dissatisfaction surrounding pharmacogenetics may originate from the belief that it will guarantee improved treatment outcomes. To mitigate disappointment, healthcare professionals and researchers should continue to emphasize that genetic profiles are just one factor, among many (e.g., age, concomitant medications), influencing medication response, not a guarantee of the ‘correct’ medication or dose. Moreover, it is important to communicate that pharmacogenetics also works alongside other paths to recovery (e.g., talking therapies).

### 4.6 Future research

Future research could build on this pilot survey by developing a validated measure of patient perspectives, enabling systematic comparisons across settings and populations. Within this, recording reasons for non-participation in such surveys is necessary to better understand barriers to engagement. Moreover, surveys with larger sample sizes are needed to compare perceptions of pharmacogenetics between sub-groups. For example, there are different distributions of CYP alleles between different world populations, thus, with a large and diverse sample, future research could investigate whether patient perceptions of pharmacogenetics vary across different ethnicity groups. For example, the CYP2D6*10 allele, which leads to reduced enzyme functionality, is almost exclusively seen in African, South Asian, and East Asian populations, with an especially high frequency (58.7%) in East Asians (Zhou et al., 2017). Researchers could investigate whether having more carriers of actionable genetic variants leads to differences in perceptions of pharmacogenetics and in the cost-effectiveness of testing. This will address a broader issue in research, particularly in pharmacogenetics, where diversity is often lacking (Virelli et al., 2023; Liko et al., 2020).

Future research should explore further whether and how pharmacogenetic testing promotes more collaborative treatment discussions. Measuring patients’ perceived involvement in medication-related decisions and trust in healthcare providers before and after pharmacogenetic testing would allow for meaningful comparisons. There are suggestion in our study that discussions of pharmacogenetic reports may support more collaborative forms of medication decision-making and enhance patients’ sense of agency. Further work is needed to investigate how test reports can best be shared in clinical consultations to enhance these potential benefits that may also strengthen therapeutic alliances and clinical engagement.

## 5 Conclusion

Both patients and clinicians in this study found the use of pharmacogenetic testing to inform antipsychotic prescribing acceptable, suggesting it may enhance medicine optimization and precision psychiatry. Further research with larger and diverse samples is required to gather broader opinions. This study adds to the growing evidence of positive patient and clinician perspectives on pharmacogenetics, supporting its use in mental healthcare.

## Data Availability

The raw data supporting the conclusions of this article will be made available by the authors, without undue reservation.
