# Supplementary material for: Patient and clinician perspectives on pharmacogenetic testing for antipsychotics
Source: Front Pharmacol. 2025 Oct 9;16:1689300. doi: 10.3389/fphar.2025.1689300 (PMC12547232; doi:10.3389/fphar.2025.1689300)
Supplement: Supplementary file 1 [file Supplementaryfile1.docx]

Supplementary Material

**A. Questions for Clinician Interview**

1. How many pharmacogenetics reports have you seen?

How many of these reports are from people who are not normal metabolisers of antipsychotics?

Have you used reports to discuss prescribing with patients?

“I’d like to focus on these variant cases for the following questions”

2. Can you tell me how you used the report in the consultation you had with the patient?

• When discussing with patients, was it easy to convey information?

• Optional probes for the main question if details remain unclear:

–  What happened (e.g., verbal explanation? Handing the report to the patient?)

–  How much time was spent on the consultation?

–  How did you decide how you were going to discuss it?

–  Reasoning behind that: Why did you choose to discuss it in this way?

–  Style of providing information and why.

–  There are many ways you could have discussed it; why did you choose this particular one?

3. For the cases with non-normal metabolism shown in the report, did it lead to any changes in your prescribing?

Why or why not?

Can you give an example of any instances when having read the pharmacogenetics report changed your prescribing decision for a patient?

4. How have the patients tended to respond to the use of a pharmacogenetic report in your consultations?

Optional probes:

- Does having a pharmacogenetics report change patients’ views on taking antipsychotics?
- Did the report change the style of interaction between you and the patients?
- Is there anything else you noticed about the interaction with patients that’s different from before when you hadn’t had pharmacogenetics reports?
- Any specific questions/comments from patients that you can recall?
- Any negative response from patients or negative changes to your interaction?

5. Based on your experience of using pharmacogenetics reports so far, has it had any impacts on patients’ treatment outcomes?

Which outcomes, in your opinion (symptom reduction, side effects, social functioning, quality of life, wellbeing, etc.)?

Any case examples?

6. Based on your experience of using pharmacogenetics reports, what do you think is the value of using this report?

Value for prescribing clinicians?

Value for patients?

7. Do you think it would be feasible for pharmacogenetics reports like this to become routine in the NHS for antipsychotics prescribing?

Optional probes:

- Do you think there would be any barriers?
- What would be needed for this to become routine?
- What kind of institutional and educational support do you need to make it more feasible?

8. Do you have anything else you would like to add about what we talked about?

9. Is there anything to add about this broad area of pharmacogenetics reports for antipsychotics prescribing that we haven’t covered in this interview?

End of questions.

**B. Participant Survey**

1. What made you decide to take part in the Genetics and Environment in Mental health

Study (GEMS)?

2. Do you feel that you are well informed about the medications that you have been given in the past 5 years?

(1 = not informed at all; 10 = very much informed)

3. How concerned are you about the long-term effects of being on medication, and the effect this might have on your general health?

(1 = not at all concerned; 10 = extremely concerned)

4. In the last 5 years how much of a say do you feel you’ve had in the decisions made about the medication prescribed to you?

(1 = not at all involved; 10 = very much involved)

5. Do you think this kind of genetic testing can help improve with joint decisions about medications?

[Very much so / slightly / Not sure / not much / not at all]

6. Did you ask your clinician for a copy of the genetic report?

[yes/no]

If YES, please continue to question 7 and 8.

If NO, please move to question 9.

7. Why was having a copy of the genetic report important to you?

8. If you had a copy of the results from your genetic test, how easy was this to understand?

(1 = did not understand at all; 10 = fully understood)

9. Did the genetic results lead to any change in medication prescribed to you or the dose you take?

[yes/no]

10. Would you like to tell us more about this?

11. If a change was made, how was this done?

12. How much of a say did you feel you had in the change made to your medication because of the genetic test results?

(1 = not at all involved; 10 = very much involved)

13. How do you feel about the change?

[Very happy / reasonably happy / neutral / not that happy / Very unhappy]

14. Was the discussion with the clinician about your genetic results easy to understand?

(1 = did not understand at all; 10 = fully understood)

15. Was the communication from the study team clear and easy to understand?

(1 = not at all; 10 = very much so

16. Did you feel that you had the right amount of detail about the study?

[Strongly disagree / slightly disagree / neutral / slightly agree / strongly agree]

17. What do you feel are the main barriers for people when using this type of genetic testing?

18. Do you have anything else you would like to mention?
